# Preoperative Vaginal Microbiome as a Predictor of Postoperative Urinary Tract Infection

**DOI:** 10.21203/rs.3.rs-4069233/v1

**Published:** 2024-04-12

**Authors:** John A. OCCHINO, Jenifer N. BYRNES, Pei-Ying Wu, Marina R. WALTHER-ANTONIO, Jun CHEN

**Affiliations:** Mayo Clinic; Mayo Clinic; National Cheng Kung University Hospital, National Cheng Kung University; Mayo Clinic; Mayo Clinic

## Abstract

This is a single Institute, prospective cohort study. We collected twenty- two postmenopausal women with pelvic organ prolapse planning to undergo vaginal hysterectomy with transvaginal pelvic reconstructive surgery, with or without a concomitant anti-incontinence procedure. Vaginal swabs and urine samples were longitudinally collected at five time points: preoperative consult visit (T1), day of surgery prior to surgical scrub (T2), immediately postoperative (T3), day of hospital discharge (T4), and at the postoperative exam visit (T5). Women experiencing urinary tract infection symptoms provided a sample set prior to antibiotic administration (T6). Microbiome analysis on vaginal and urinary specimens at each time point. Region V3-V5 of the 16S ribosomal RNA gene was amplified and sequenced. Sample DNA was analyzed with visit T1, T2, T5 and T6. Six (27.3%) participants developed postoperative urinary tract infection whose vaginal sample at first clinical visit (T1) revealed beta-diversity analysis with significant differences in microbiome structure and composition. Women diagnosed with a postoperative urinary tract infection had a vaginal microbiome characterized by low abundance of Lactobacillus and high prevalence of Prevotella and Gardnerella species. In our cohort, preoperative vaginal swabs can predict who will develop a urinary tract infection following transvaginal surgery for pelvic organ prolapse.

## Introduction

Postoperative complications, including urinary tract infection, negatively impact patient care and are a substantial cost and burden to the healthcare system. in the United States, urinary tract infections account for more than 8.1 million visits to health care providers, costing an estimated $1.6 billion annually.^1,2^ Risk factors for urinary tract infection include female sex, advancing age, and surgery for pelvic organ prolapse and urinary incontinence. Voiding dysfunction as a result of pelvic organ prolapse surgery significantly increases the likelihood of developing a urinary tract infection after hysterectomy with reconstructive surgery, and 50% of postmenopausal women with anterior wall prolapse and an elevated postvoid residual greater than 150mL developed a postoperative urinary tract infection in one study.^3^ Addressing postoperative urinary tract infection is of paramount importance given the clinical burden.

It is widely believed that a healthy and balanced microbiome exerts a positive influence on human health while an unhealthy one can lead to inflammation and infection.^4^ The vaginal microbiome has been growing as an area of research with demonstrated impact in women’s health including: urinary incontinence^5^ and preterm birth.^6,7^ Although the lower urinary tract was previously thought to be sterile, the female urinary microbiome exists and impacts lower urinary tract symptoms.^8^ Recent research in the female urinary microbiotain women with urgency urinary incontinence have demonstrated an association between microbial diversity and composition and symptom severity and treatment response.^9^ Additionally, women enrolled in a clinical trial comparing anticholinergic medication and intradetrusor injection of OnabotulinumtoxinA^10^ who experienced a posttreatment urinary tract infection had fewer *Lactobacillus* species than those who did not develop urinary tract infections. Little is known about the changes that occur in microbiome of the vagina or lower urinary tract in the perioperative period as they relate to postoperative urinary tract infection.

The objective of this study was to characterize the vaginal and urinary microbiome in a cohort of postmenopausal women undergoing vaginal hysterectomy with transvaginal pelvic reconstruction for pelvic organ prolapse, with or without a concomitant anti-incontinence procedure. We sought to elucidate the stability of the microbial community and identify changes that might predict the development of postoperative urinary tract infection. Perioperative longitudinal sample collection was utilized to understand the pervasiveness of the microbes in the vagina and lower urinary tract, how they change as a result of surgery, and to identify particular microbes that may be associated with postoperative urinary tract infection.

## Results

### Patient demographics and clinical treatment course

Twenty-five postmenopausal women undergoing vaginal hysterectomy with pelvic reconstructive surgery were enrolled between May 1, 2016 and May 31, 2017. One patient was excluded for antibiotic use within 2 weeks of her planned surgery date, and two declined participations on the day of surgery, leaving 22 participants eligible for analysis. The median age was 71 years and median BMI was 26.9. All patients were Caucasian and self-identified as heterosexual. All patients were vaginally parous, with a median parity of 3. Preoperatively, 2 patients utilized a pessary (9.1%). Fifteen women reported urinary incontinence (68.2%). Thirteen women (59.1 %) did not use any form of estrogen replacement therapy. Two women (9.1%) reported a history of oral hormone replacement therapy, and 7 (31.8%) used vaginal estrogen cream perioperatively.

All patients underwent vaginal hysterectomy with native tissue apical suspension, cystoscopy and concomitant pelvic reconstruction as indicated. The clinical characteristics in UTI and non-UTI groups were similar (Suppplementary table 1). All surgical pathology was benign. Five (22.7%) women underwent concomitant retropubic synthetic (mesh) midurethral sling for treatment of stress urinary incontinence. Anticipated postoperative voiding dysfunction was managed with suprapubic catheter or clean intermittent catheterization based on surgeon preference. Eleven (50%) patients had a suprapubic catheter placed at the time of surgery and 11 (50%) underwent a voiding trial prior to hospital dismissal. The median duration of suprapubic catheter use was 10 days (range 7.5–30 days). Six women failed the postoperative voiding trial, of which 5 (22.3%) performed self-intermittent catheterization (SIC) and 1 (4.5%) was managed with a transurethral Foley catheter. One woman required surgical revision of her midurethral sling for persistent voiding dysfunction two weeks after her initial surgery for prolapse.

### Urine bacteria culture data in urinary tract infection group

Six (27.3%) women were diagnosed with a postoperative urinary tract infection, all of whom had postoperative voiding dysfunction (2 SIC, 3 suprapubic catheter, 1 office evaluation for urinary hesitancy). Three of these patients underwent concomitant midurethral sling. They performed self-intermittent catheterization (SIC) for one day and fourteen days. Both standard urine culture and microbiome data was available. Urine cultures were positive for *Escherichia coli* (2), *Pseudomonas aeruginosa* (1), and *Enterobacter agglomerans* (1). Two women outside our institution were unable to obtain a urine culture and were treated empirically with antibiotics for presumed UTI.

### Different vaginal microbiome in the non-UTI group and the UTI group in the first clinical examination

**Figure 1:**
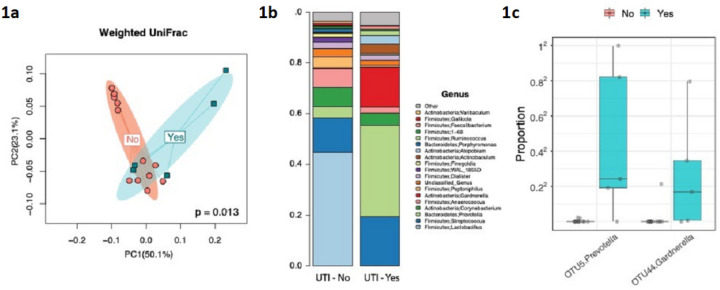
Vaginal swab stratified by urinary tract infection.

DNA was able to be analyzed for samples from visit T1, T2, T5 and T6, but was insufficient for analysis for T3 (immediately after surgery) and T4 (prior to hospital discharge). Alpha-diversity analysis did not reveal a significant effect for the measures investigated for both the vaginal and urine samples. However, β-diversity analysis showed a significant compositional difference for the vaginal swab sample from the first clinical visit (T1) in patients diagnosed with a urinary tract infection (Table 1, Figure 1A). The results were significant for all β-diversity measures, indicating a potentially strong microbial signature in the vaginal microbiome for patients who developed a postoperative urinary tract infection. A similar trend was noted for the urinary microbiome, but this did not reach statistical significance. Women diagnosed with a postoperative urinary tract infection had a vaginal microbiome characterized by low abundance of *Lactobacillus* and high prevalence of *Prevotella* and *Gardnerella* (Figure 1B). Two OTUs, the *Prevotella* OTU5 and *Gardnerella* OTU44, were significantly associated with UTI in T1 (Figure 1C). Although these OTUs were not significant after multiple testing correction for V2 and V5, a trend of high prevalence with urinary tract infection was still observed in the vaginal swab (Supplementary Figure 1).

### Different microbiome data in vaginal swap and urine samples

**Figure 2:**
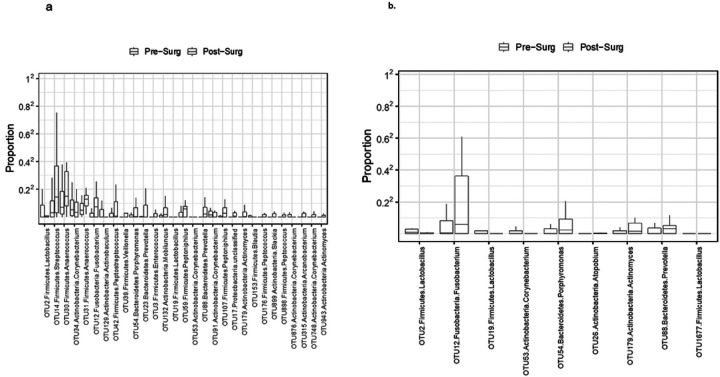
a.Surgical effect on the vaginal microbiome. b. Surgical effect on the urine microbiome.

We also analyzed the effect of surgery on the vaginal and urinary microbiome by comparing the preoperative vaginal swabs and urine samples (T1 and T2) to the postoperative (T5) vaginal swabs and urine samples. Alpha-diversity analysis based on Shannon index revealed that surgery increased the overall diversity in the vaginal microbiome (p = 0.002, Shannon index), but that was not observed in the urinary microbiome. B-diversity analysis showed a significant effect of surgery in the overall microbiome composition for both the vaginal and urinary microbiome (Table 2). Differential abundance analysis identified a large number of taxa associated with the surgical effect for both vaginal swabs (Figure 2A) and urine samples (Figure 2B).

## Discussion

We sought to prospectively characterize the vaginal and urinary microbiome longitudinally to investigate the changes that occur in the perioperative period as related to surgery and postoperative infection. Preoperative vaginal microbiome may be associated with postoperative urinary tract infections following pelvic reconstruction surgery. Low *Lactobacillus* abundance and high prevalence of *Prevotella* and *Gardnerella* species may contribute to the development of post-surgery infections. Furthermore, the results are consistent with those reported by Thomas-White, who concluded that post-operative UTIs were caused by reduced Lactobacillus spp compared to non-operative UTI.^11^ In our study, the characteristics of the patient did not play a major role in the development of postoperative urinary tract infections. Menopause, age, and comorbidities were not significant contributing factors to postoperative urinary tract infections. UTI has been found to be associated with a PVR greater than 150 ml, prolapse of the anterior vaginal wall and operative cystoscopy in PFR surgery, according to a retrospective study.^3^ Our study did not find that these factors were predisposing in our patient cohort

A previous study found *Lactobacillus* spp. (CST I) to be the most prevalent vaginal microbiome type in postmenopausal women, followed by CST VI-A *(non-Lactobacillus spp)*.^12^ Studies investigating the female urinary microbiome have identified various urotypes based on the predominant taxon identified in the sample.^13^In menopausal women, less *Lactobacillus* species are known to be a predisposing factor for UTIs. And while the vaginal microbiome has been found to be similar to the bladder microbiome, most studies indicate that UTIs are associated with the vaginal microbiome in most cases.^14,15^ Our data is aligned with this finding. Women with a vaginal microbiome with a low abundance of Lactobacillus tended to diagnose with a postoperative urinary tract infection. Several papers have also demonstrated a protective effect of Lactobacillus.^13,14,16^

Positive day of surgery urine cultures have been associated with an increased risk of postoperative urinary tract infection with nearly 30% of women experiencing infection within 6 weeks of surgery.^17^ Ideally, we would be able to identify women at risk for urinary tract infection sufficiently early to implement changes to decrease this risk. In our study, preoperative vaginal swabs collected at the initial surgical consult visit were significantly different in women who developed a postoperative urinary tract infection. Although preliminary, our data suggest women could be screened at the surgical consultation visit, which would provide adequate time to implement changes to modify the risk.

Estrogen influences the vaginal microbiome^18^ and studies have also demonstrated the effect of estrogen on the female urinary microbiome.^19^ Loss of predominance by a single microbe like *Lactobacillus* in the bladder has been associated with urgency urinary incontinence symptoms, and lower urinary tract symptoms like urgency and frequency can be treated with vaginal estrogen therapy. We chose to study a cohort of postmenopausal women to reduce the effect of endogenous hormone fluctuations on the urogenital microbiome. We did not limit the use of oral or vaginal estrogen replacement therapy in this study, and more than half of the study participants (59%) did not use any hormone replacement therapy. Approximately 30% of women reported using vaginal estrogen cream postoperatively and self-reported usage did not differ in women who developed a postoperative urinary tract infection.

This study has several strengths, most notably the prospective, longitudinal collection of both vaginal swabs and urine samples. Additionally, next generation sequencing techniques and analysis provide a comprehensive evaluation of the baseline vaginal and urinary microbiomes in our cohort of women undergoing vaginal hysterectomy and pelvic reconstruction and can detect changes associated with surgery and urinary tract infection. There are limitations to our study. This is a pilot study and there are only 22 patients recruited within 6 patients of post-operation UTI. The generalizability of our results is limited by the lack of ethnic diversity as all patients in this study were Caucasian. Two of the women in our study were empirically treated with antibiotics without urine culture results; although this is a common practice, it limits our ability to compare standard urine culture results to the microbiome analysis for these participants. Preoperative urine samples and those collected for evaluation of symptomatic urinary tract infection were collected by clean catch, which could potentially be affected by vulvovaginal contamination, although other studies have utilized similar methodology.^14,19^ Future studies may utilize catheter collected urine specimens to avoid the possibility of vulvovaginal contamination. In our study, perioperative longitudinal evaluation samples collected immediately after surgery (T3) and prior to hospital discharge (T4) did not contain adequate DNA for analysis. The lack of adequate DNA in the immediate postoperative vaginal swab and urine sample was likely the result of dilution introduced by cystoscopy fluid during surgical procedure and vaginal betadine preparation. The use of preoperative intravenous antibiotics may have contributed to the lack of DNA in the samples collected on the day of hospital discharge, as all patients were discharged on the day after surgery. This suggests disruption of the vaginal and urinary tract microbiome may persist for several days following induced changes. Future studies investigating longitudinal changes in the vaginal and urinary microbiome could eliminate these collection points. Finally, a larger study powered to detect differences in urotypes may improve the ability to identify women at risk for urinary tract infection through a urine sample.

This prospective cohort study was designed to guide future studies on the vaginal and urinary microbiome. Longitudinal evaluation of the vaginal and urinary microbiome is feasible. The clinical impact of our findings is significant, as we could predict women for postoperative urinary tract infection with a preoperative vaginal swab. Confirming these findings through larger studies with ethnical diversity could ultimately allow us to screen women before surgery, individualize our preoperative counseling on surgical risks, and possibly delay surgery until treatment is completed.

## Materials And Methods

### Subject Recruitment

Patients’ recruitment following *Mayo Clinic institutional board review approval (#15–006300)* 25 patients evaluated in the Division of Urogynecology with symptomatic pelvic organ prolapse planning to undergo pelvic reconstructive surgery were approached for study participation. All patients were obtained informed consent from themselves before joining the trial. All experiments were performed in accordance with Mayo Clinic institution guidelines and regulations. Eligible participants were postmenopausal, planning transvaginal surgical correction of pelvic organ prolapse that included vaginal hysterectomy with concomitant native tissue pelvic reconstruction and cystoscopy, a scheduled surgery date within 4 weeks of study enrollment, and were physically able to collect clean-catch urine samples as well as self-collected vaginal swabs. Concomitant anti-incontinence surgery was allowed. Women with any of the following criteria were excluded: currently taking or had taken antibiotics in the past 2 weeks, history of recurrent urinary tract infections, and a history of mesh complications, including erosion/extrusion. We did not exclude women with postoperative urinary retention or voiding dysfunction requiring either a suprapubic catheter or clean intermittent self-catheterization. Participants completed an optional questionnaire about sexual and reproductive health and history.

### Sample Collection

A sample set included 1 vaginal swab and 1 urine sample (Supplementary figure 2). Each set was collected at five time points: preoperative surgical consult visit (T1), day of surgery prior to the standard pre-operative surgical scrub (T2), immediately following surgery completion (T3), day of hospital discharge (T4), and at the postoperative exam visit 5–8 weeks following surgery (T5). Samples for T1, T2, T3, T4, and T5 were collected by a member of the research team. An additional set of samples was obtained from women experiencing symptoms of a urinary tract infection prior to antibiotic administration (T6). The vaginal swabs were collected by pressing and rotating 2 sterile Puritan swabs along the lateral vaginal walls approximately 3” inside the introitus for at least 5 seconds and placing them in a sterile Aptima tube. Clean-catch midstream urine samples were collected by the patient in a sterile urine cup container after instruction by the research coordinator.

Participants experiencing symptoms of a urinary tract infection within 6 weeks of surgery were instructed to schedule an appointment with a provider or visit an urgent care center for evaluation and urine culture. Symptomatic urinary tract infection was defined using the CDC criteria as described below.^20^

- Patients with one of the following:
  - Fever (> 38°C or 100.4°F), frequency, dysuria, suprapubic tenderness, costovertebral angle pain or tenderness
  - AND: a urine culture of >100,000 colonies/mL with no more than 2 species of organisms.
- Patients with two of the following criteria:
  - Fever (> 38°C or 100.4°F), urgency, frequency, dysuria, suprapubic tenderness, costovertebral angle pain or tenderness
  - AND at least one of the following:
    1. Dipstick test positive for leukocyte esterase and/or nitrate
    2. Pyuria (> 10 WBCs/mm^3^ or >3 WBC/hpf of unspun urine)
    3. Organisms seen on gram stain of unspun urine
    4. 2 urine cultures with <100,000 colonies/mL urine of single uropathogen in non-voided specimen.
    5. Urine culture with <100,000 colonies/mL of a single uropathogen in a patient being treated with appropriate antimicrobial therapy.
    6. Physician’s diagnosis
    7. Physician institutes appropriate antimicrobial therapy

#### Bacterial DNA extraction

The methodological procedures have already been developed and implemented by Mayo Clinic and others.^21^ The bacterial cells were harvested from the broth solution. Microbial DNA was extracted using BiOstic Bacteremia DNA Isolation Kit (MoBio Laboratories, Inc., Carlsbad, CA), according to manufacturer’s instructions. Microbial DNA was quantified using Qubit Fluorometric quantitation (ThermoFisher Scientific, Waltham, MA). The resulting DNA was stored at −80°C.

### PCR Primers and Probe Design

The microbial 16S rRNA gene was amplified through a polymerase chain reaction (PCR) in the following working solution: 10μL of 2x Taqman Universal Master Mix II (AmpliTaq Gold DNA polymerase, deoxynucleotide triphosphates, ROX^™^ Passive Reference dye, optimized buffer components; ThermoFisher Scientific, Grand Island, NY), 2μL (9μM) forward primer, 2μL (9μM) reverse primer, 2μL (2.5μM) probe, 2μL sample DNA, and 2uL molecular grade water up to a final volume of 50μL per reaction.

V3_357F and V5_926R primers^22^ modified with Nextera adaptors were developed in collaboration with the University of Minnesota Genomic Center in Minneapolis, MN.

V3_341 F_Nextera:

TCGTCGGCAGCGTCAGATGTGTATAAGAGACAGCCTACGGGAGGCAGCAG

V5_926R_Nextera:

GTCTCGTGGGCTCGGAGATGTGTATAAGAGACAGCCGTCAATTCMTTTRAGT

The RT-PCR standard universal thermal cycling protocol was performed using the QuantStudio^™^ 6 Flex (ThermoFisher Scientific) as follows: activation of DNA polymerase at 50°C for 2 minutes then increased to 95°C for 10 minutes followed by 40 cycles of denaturation at 95°C for 15 seconds followed by annealing/extension phase at 60°C for 1 minute. Data collection occurred during the annealing/extension phase. Samples were plated in 384 well plates and covered with optical adhesive. Plates centrifuged at 2000 RPM for 2 minutes to remove bubble from bottom of wells. All samples were run in triplicate with positive and negative controls.

### Bioinformatics and Statistical Analysis

The clinical characteristics of all participants compared UTI and non-UTI groups. Independent t test was used for continuous variables and Fisher exact test for categorical variables. After paired-end sequencing, the bioinformatics pipeline *hybrid-denovo*^23^ was used to cluster the paired- end reads into operational taxonomic units (OTUs) at 97% similarity level. OTUs were assigned taxonomy using the Ribosomal Database Project Classifier and the GreenGenes database (v13.5).^24^ FastTree algorithm was used to construct a phylogenetic tree based on the OTU representative sequences.^25^ Singleton OTUs were removed in the pipeline. We further removed OTUs highly represented in the negative controls and discarded samples with less than 1,000 reads.

After these quality control steps, a total of 133 samples with 2,955,441 high-quality reads and 780 OTUs were analyzed. The sequencing depth of the samples ranged from 1,010 to 53,874 with a median of 23,088. The detected OTUs belong to 15 phyla, 84 families and 157 genera. OTU data were also summarized into α-diversity and β-diversity after rarefaction (1,010 reads per sample). Alpha-diversity (within-sample diversity) reflects species richness and evenness within the microbial community. Two α-diversity indices were calculated: observed OTU number, a species richness measure, and Shannon index, an overall measure considering both richness and evenness. Beta-diversity (between-sample diversity) reflects the shared diversity between microbial communities in terms of ecological distance; pair-wise distance measure allows quantification of the overall compositional difference between samples. Different α-diversity measures provide distinctive views of the community structure. Thus by investigating multiple β-diversity measures it allows us to assess the extent and the nature of compositional difference. Three β-diversity measures, unweighted, generalized (α=0.5) and weighted UniFrac distances, were calculated using the OTU data and a phylogenetic tree (“GUniFrac” function in the R package “GUniFrac”).^26^ The unweighted UniFrac captures differences in community membership while the weighted/generalized UniFrac captures differences in abundances. Generalized UniFrac reduces the weights on the abundant lineages and is more powerful to reveal the community difference in less abundant lineages.^26^ Bray-Curtis distance, which does not depend on the phylogenetic tree, was also calculated (with the “vegdist” in the R package “vegan”) to capture potential phylogenetically non-related community difference.

#### Urinary Tract Infection Analysis

To test the association between the urinary tract infection status (covariate) and α-diversity (outcome), simple linear regression was used. To test the overall compositional difference between the urinary tract infection status based on β-diversity, PERMANOVA, a distance-based analysis of variance method based on permutation (“adonis” function in the R “vegan” package),^27^ was used. Ordination plots were generated using the first two principal coordinates from PCoA (Principal Coordinate Analysis) based on the β-diversity distance matrices (“cmdscale” function in the R). Differential abundance analysis was performed at various taxonomical ranks, and taxa with prevalence less than 10% or with a maximum proportion less than 0.2% were excluded from testing to reduce the number of the tests. The count data was first normalized into relative abundances by the GMPR(Geometric Mean of Pairwise Ratios) size factor.^28^ To identify specific taxa associated with urinary tract infection, Wilcoxon rank sum test was used. False discovery rate (FDR) control (BH procedure, “p.adjust” in R) was performed to correct for multiple testing.^29^ Taxa with an FDR-adjusted p-value or q-value less than 0.2 were considered to be significant (i.e, we expect an average of 20% false positives in the results). The urinary tract infection association analyses were performed for each visit (T1, T2, T5) and for vaginal swab and urine samples separately.

#### Surgical Effect Analysis

To detect the surgical effect on the vaginal and/or urinary microbiome by comparing T1/T2 to T5, similar analyses were performed on the α-diversity, β-diversity and taxon-level data. To account for the within-subject correlation, a linear mixed effects model was used for α-diversity analysis, and within-subject permutation was used for β-diversity. To identify specific taxa associated with surgical effect, a permutation-based approach (within-subject permutation) based on the F-statistics of a linear model with square-root transformed relative abundance as the outcome was used.^30^ Differential taxa were identified with an FDR-adjusted p-value or q-value less than 0.2. All statistical analyses were performed in R 3.3.2 (R Development Core Team).

## Figures and Tables

**Table 1:** Testing the microbiome compositional differences between UTI(+) and UTI (−) groups based on b-diversity measures.

	Unweighted UnifFrac	Generalized UniFrac	Weighted UniFrac	Bray-Curtis
Swab T1 [R^2^]	0.092	0.138	0.222	0.148
P value	**0.049**	**0.025**	**0.013**	**0.033**
Swab T2 [R^2^]	0.065	0.076	0.092	0.149
P value	0.197	0.18	0.149	**0.026**
Swab T5 [R^2^]	0.079	0.07	0.071	0.117
P value	0.186	0.37	0.356	**0.043**
Urine T1 [R^2^]	0.067	0.087	0.112	0.127
P value	0.341	0.212	0.18	0.172
Urine T2 [R^2^]	0.04	0.083	0.126	0.148
P value	0.71	0.158	0.057	**0.027**
Urine T5 [R^2^]	0.05	0.044	0.047	0.059
P value	0.525	0.613	0.532	0.382

R^2^, the percent variability explained, is a measure of the effect. PERMANOVA test is used

**Table 2:** Testing the surgical effect on the overall microbiome composition based on b-diversity measures.

	Unweighted UniFrac	Generalized UniFrac	Weighted UniFrac	Bray-Curtis
Vaginal Swab [R^2^]	0.033	0.043	0.032	0.045
P value	**0.005**	**0.004**	**0.026**	**0.003**
Urine Sample [R^2^]	0.017	0.027	0.036	0.057
P value	0.107	**0.042**	**0.017**	**0.001**

R^2^, the percent variability explained, is a measure of the effect. PERMANOVA test is used

## Data Availability

The 16s rRNA sequences data was uploaded in NCBI SRA database. The BioProject ID was PRJNA1077395. The datasets also can be available from the corresponding author on reasonable request.
